# Norepinephrine and dopamine increase motility, biofilm formation, and virulence of *Vibrio harveyi*

**DOI:** 10.3389/fmicb.2014.00584

**Published:** 2014-11-06

**Authors:** Qian Yang, Nguyen D. Q. Anh, Peter Bossier, Tom Defoirdt

**Affiliations:** Laboratory of Aquaculture and Artemia Reference Center, Department of Animal Production, Ghent UniversityGhent, Belgium

**Keywords:** swimming motility, flagellum, virulence, shrimp, antivirulence therapy, microbial endocrinology

## Abstract

*Vibrio harveyi* is one of the major pathogens of aquatic organisms, affecting both vertebrates and invertebrates, and causes important losses in the aquaculture industry. In order to develop novel methods to control disease caused by this pathogen, we need to obtain a better understanding of pathogenicity mechanisms. Sensing of catecholamines increases both growth and production of virulence-related factors in pathogens of terrestrial animals and humans. However, at this moment, knowledge on the impact of catecholamines on the virulence of pathogens of aquatic organisms is lacking. In the present study, we report that in *V. harveyi*, norepinephrine (NE) and dopamine (Dopa) increased growth in serum-supplemented medium, siderophore production, swimming motility, and expression of genes involved in flagellar motility, biofilm formation, and exopolysaccharide production. Consistent with this, pretreatment of *V. harveyi* with catecholamines prior to inoculation into the rearing water resulted in significantly decreased survival of gnotobiotic brine shrimp larvae, when compared to larvae challenged with untreated *V. harveyi*. Further, NE-induced effects could be neutralized by α-adrenergic antagonists or by the bacterial catecholamine receptor antagonist LED209, but not by β-adrenergic or dopaminergic antagonists. Dopa-induced effects could be neutralized by dopaminergic antagonists or LED209, but not by adrenergic antagonists. Together, our results indicate that catecholamine sensing increases the success of transmission of *V. harveyi* and that interfering with catecholamine sensing might be an interesting strategy to control vibriosis in aquaculture. We hypothesize that upon tissue and/or hemocyte damage during infection, pathogens come into contact with elevated catecholamine levels, and that this stimulates the expression of virulence factors that are required to colonize a new host.

## INTRODUCTION

*Vibrio harveyi* is a ubiquitous, bioluminescent marine bacterium which can cause luminous vibriosis in both marine vertebrates and invertebrates, leading to significant losses in the global aquaculture industry (Austin and Zhang, 2006; Ruwandeepika et al., 2012). For example, it has been reported to be associated with high mortalities of cultured penaeid shrimp larvae and packhorse rock lobster larvae (Soto-Rodriguez et al., 2003), diseased sea horses (Tendencia, 2004), and skin ulceration in juvenile sea cucumber (Becket et al., 2004). The pathogenicity of *V. harveyi* is considered to involve biofilm formation, swimming motility, and the production of various extracellular products, such as hemolysins, proteases, (phospho)lipases, and chitinases (Karunasagar et al., 1994; Austin and Zhang, 2006; Ruwandeepika et al., 2012; Yang and Defoirdt, 2014). Conventional antibiotics are becoming increasingly ineffective to control bacterial infections in aquaculture, and consequently, alternative methods to control infections are urgently needed (Defoirdt et al., 2011). In this respect, inhibiting the production of virulence-related phenotypes, a strategy that has been termed antivirulence therapy, is an interesting novel approach for controlling bacterial infections (Clatworthy et al., 2007; Defoirdt, 2013, 2014). The inhibition of specific virulence genes is possible (Baron, 2010). However, much more research effort thus far has been devoted to virulence-regulatory mechanisms because these mechanisms control the expression of (multiple) virulence factors and consequently, by targeting these mechanisms it would be possible to block several virulence factors at once.

Host stress has long been known to influence host–pathogen interactions. For a long time, the impact of stress on infection has been exclusively associated with the suppression of the immune system of the host and an increased susceptibility to infections due to elevated levels of stress hormones (Dhabhar and McEwen, 1997; Freestone et al., 2008). However, investigations over the past decades have introduced a new perspective which implies that infectious bacteria also respond to these stress hormones (Lyte, 2004). The catecholamine stress hormones norepinephrine (NE) and dopamine (Dopa), which are an integral part of the ‘fight or flight’ stress response in animals (Reiche et al., 2004), stimulate the growth of several species of bacteria (including *Escherichia coli*, *Yersinia enterocolitica*, *Listeria monocytogenes*, *Aeromonas hydrophila*, *Pseudomonas aeruginosa,* and *V. parahaemolyticus*) in serum-based media (Lyte and Ernst, 1992; Coulanges et al., 1997; Kinney et al., 1999; Belay et al., 2003; Nakano et al., 2007a,b). Such media are iron-limited because of chelation of free iron by high-affinity iron-binding proteins such as transferrin. According to Sharaff and Freestone (2011), catecholamines increase the availability of iron through complex formation with the iron-binding proteins, thereby decreasing their affinity for iron to a point of iron loss. Catecholamines have also been reported to increase the production of Shiga toxin, chemotaxis, biofilm formation, and attachment and colonization to epithelial cells in pathogenic *E. coli*, motility and invasiveness of *Campylobacter jejuni*, motility and type III secretion in *Salmonella typhimurium*, and cytotoxic activity in *V. parahaemolyticus* (Bansal et al., 2007; Cogan et al., 2007; Nakano et al., 2007b; Lyte et al., 2011; Sharaff and Freestone, 2011).

Catecholamines exert their effects by binding to specific receptors. In eukaryotes, epinephrine and NE bind to adrenergic receptors, which are divided into two major families (α and β), each with a number of receptor subtypes, while Dopa binds to dopaminergic receptors with at least five receptor subtypes; and binding of the hormones to the receptors can be prevented by specific antagonists (Freestone et al., 2007). Interestingly, antagonists of eukaryotic adrenergic and dopaminergic receptors can also inhibit catecholamine-induced effects in bacteria (Sharaff and Freestone, 2011). Furthermore, several bacterial catecholamine receptors have been reported as well, including the histidine sensor kinases QseC and QseE (Hughes et al., 2009), for which a highly active antagonist, LED209, has been identified (Rasko et al., 2008).

The production of catecholamines is highly conserved in the animal kingdom, both in vertebrates (including fish) and invertebrates (including mollusks and crustaceans; Ottaviani and Franceschi, 1996). However, at this moment, knowledge on the impact of catecholamines on the virulence of major pathogens of aquatic organisms, such as *V. harveyi,* is lacking. In this study, we aimed at investigating the impact of the catecholamines NE and Dopa on the growth of *V. harveyi* in serum-based medium, on the expression of various virulence-related characteristics and on virulence toward gnotobiotic brine shrimp (*Artemia franciscana*) larvae.

## MATERIALS AND METHODS

### BACTERIAL STRAINS AND GROWTH CONDITIONS

*Vibrio harveyi* wild type strain ATCC BAA-1116 (recently reclassified as *V. campbellii*; Lin et al., 2010) was used in this study. Unless otherwise stated, the strain was cultured at 28°C in Luria broth containing 35 g/L sodium chloride (LB_35_) under constant agitation (100 min^-1^). Cell densities were measured spectrophotometrically at 600 nm.

### CATECHOLAMINES AND EUKARYOTIC CATECHOLAMINE RECEPTOR ANTAGONISTS

Norepinephrine was dissolved in hydrochloride acid (HCl 0.1 N) at 10 mM, while Dopa was dissolved in distilled water at 10 mM. The antagonists used in this study are listed in **Table 1**. All the chemicals were purchased from Sigma–Aldrich (Bornem, Belgium). The reagents were sterilized using a 0.22 μm filter and stored at -20°C.

**Table 1:** Catecholamine receptor antagonists used in this study.

Compound	Specificity	Solvent
Phentolamine hydrochloride	Reversible α-adrenergic	Ethanol
Phenoxybenzamine hydrochloride	Irreversible α-adrenergic	DMSO
*S*-propranolol hydrochloride	β-adrenergic	Ethanol
Labetalol hydrochloride	α- and β-adrenergic	Ethanol
Chlorpromazine hydrochloride	Dopaminergic	Distilled water
LED209^1^	Bacterial catecholamine receptor QseC	DMSO

*All compounds were dissolved at 10 mM, except for LED209, which was dissolved at 10 μM.*

*^1^*N*-phenyl-4-[[(phenylamino)thioxomethyl]amino]-benzenesulfonamide.*

### BACTERIAL GROWTH ASSAYS

For the bacterial growth assays, *V. harveyi* was grown overnight in LB_35_ broth at 28°C. After that, the culture was re-inoculated at a concentration of 10^2^ CFU/ml into fresh LB_35_ broth containing 30% (v/v) adult bovine serum (Sigma–Aldrich), with and without 50 μM NE or Dopa. Additionally, different concentrations of the catecholamine receptor antagonists were added in conjunction with the catecholamines to determine whether they could neutralize catecholamine-induced growth responses. The cultures were grown in 200 μl volumes in 96-well plates at 28°C for 48 h, and the turbidity at 600 nm was monitored every hour using a Multireader machine (Infinite M200, TECAN, Austria). Growth curves were determined for three independent cultures, and the growth rate of the exponentially growing cultures was calculated. The statistical significance of specific growth rate was determined using an independent samples *t*-test.

### SIDEROPHORE ACTIVITY ASSAY

The siderophore activity was determined by Chrome azurol S (CAS) agar diffusion assay according to Shin et al. (2001), which was performed as follows. The CAS agar plates were prepared according to Schwyn and Neilands (1987); 60.5 mg (CAS; Sigma–Aldrich) was dissolved in 50 ml deionized water, and mixed with 10 ml iron (III) solution (1 mM FeCl_3_⋅6H_2_O, 10 mM HCl). Under stirring, this solution was slowly added to 72.9 mg hexadecyltrimethylammonium bromide (Sigma–Aldrich), dissolved in 40 ml water. The resultant dark blue solution was autoclaved and stored in a plastic container. Then 100 ml 10× MM9 salts, 15 g agar, 30.24 g Pipes, and 12 g of a 50% (w/v) NaOH solution (to raise the pH to 6.8) were added to 750 ml water. After autoclaving and cooling to 50°C, 30 ml of a sterile 10% casamino acids solution was added as the carbon source. The dye solution was finally added with enough agitation to achieve mixing without generation of foam. Serum (30%, v/v), catecholamines and antagonists were added directly into the agar. *V. harveyi* was cultured overnight in LB_35_ broth containing 30% (v/v) serum, with or without catecholamines and antagonists. Then the CAS agar plates were punched with 5-mm-diameter holes and each hole was filled with 35 μl of a *V. harveyi* culture (OD_600_ = 1.0). After incubation at 28°C for 24 h, the size of the orange halo formed around each hole was measured. Siderophore activity was expressed as the square value of the halo diameter.

### SWIMMING MOTILITY ASSAY

The swimming motility assay was performed on soft agar (LB_35_ plates containing 0.3% agar) as described previously (Yang and Defoirdt, 2014). The catecholamines and antagonists were added to the autoclaved agar. *V. harveyi* was grown overnight in LB_35_ broth, and 5 μl aliquots (OD600 = 1.0) were spotted in the center of the soft agar plates. Plates were incubated for 24 h, after which the diameters of the motility halos were measured. All assays were done with freshly prepared media in six replicates.

### BIOFILM FORMATION ASSAY

Biofilm formation assay was quantified by crystal violet staining, as described previously (Stepanović et al., 2007). In brief, an overnight culture of *V. harveyi* was diluted to an OD_600_ of 0.1 in LB_35_ broth with or without catecholamines and antagonists, and 200 μl aliquots of these suspensions were pipetted into the wells of a 96 well plate. Then the bacteria were allowed to adhere and grow without agitation for 24 h at 28°C. After that, the cultures were removed and the wells were washed three times with 300 μl sterile physiological saline to remove all non-adherent bacteria. The remaining attached bacteria were fixed with 150 μl of 99% methanol per well for 20 min, after which the methanol was removed and plates were air-dried. Then, biofilms were stained for 15 min with 150 μl of a 1% crystal violet solution (Pro-lab Diagnostics, Richmond Hill, ON, Canada) per well. Excess stain was rinsed off by placing the plate under running tap water, and washing was continued until the washings were free of the stain. After the plates were air dried, the dye bound to the adherent cells was resolubilized with 150 μl of 95% ethanol per well, and absorbance was measured at 570 nm. Sterile medium served as negative control. For the quantification of exopolysaccharides, Calcofluor white staining (Sigma–Aldrich) was used. In brief, wells were rinsed after 24 h biofilm formation and 100 μl phosphate buffered saline containing 0.5 μl 5 mM Calcofluor white staining dye was added to the wells. After 60 min, fluorescence (excitation 405 nm and emission 500 nm) was measured with a Multi-reader (Infinite M200, TECAN, Austria).

### LYTIC ENZYME ACTIVITY ASSAYS

All assays were conducted according to Natrah et al. (2011). For each assay, an overnight culture of *V. harveyi* was diluted to an OD600 of 0.5 and 5 μl of the diluted culture was spotted in the middle of the test plates. The catecholamines and antagonists were added to the autoclaved agar before pouring. All assays were done at least in triplicate. Lipase and phospholipase activities were assessed on marine agar plates supplemented with 1% Tween 80 (Sigma–Aldrich) and 1% egg yolk emulsion (Sigma–Aldrich), respectively. The development of opalescent zones around the colonies was observed and the diameters of the zones were measured after 2–4 days of incubation at 28°C. Caseinase assay plates were prepared by mixing double strength Marine Agar with a 4% skim milk powder suspension (Oxoid, Basingstoke, Hampshire, UK), sterilized separately at 121°C for 5 min. Clearing zones surrounding the bacterial colonies were measured after 2 days of incubation. Gelatinase assay plates were prepared by mixing 0.5% gelatin (Sigma–Aldrich) into the agar. After incubation for 7 days, saturated ammonium sulfate (80%) in distilled water was poured over the plates and after 2 min, the diameters of the clearing zones around the colonies were measured. Hemolytic assay plates were prepared by supplementing Marine Agar with 5% defibrinated sheep blood (Oxoid) and clearing zones were measured after 2 days of incubation.

### RNA EXTRACTION

*Vibrio harveyi* was grown overnight in triplicate on soft agar plates (0.3% agar). Cells were harvested and RNA was extracted with the SV Total RNA Isolation System (Promega, Leiden, The Netherlands) according to the manufacturer’s instructions. The RNA quantity was measured spectrophotometrically (NanoDrop Technologies, Wilmington, DE, USA) and adjusted to 200 ng μl^-1^ in all samples. The RNA integrity was checked by Agarose Gel Electrophoresis and the RNA samples were stored in -80°C for subsequent use.

### PRIMERS

Specific primers were used for 10 selected genes involved in motility of *V. harveyi* (Yang and Defoirdt, 2014). The RNA polymerase A submit (*rpoA*) mRNA was used as an endogenous control (Defoirdt et al., 2007).

### REVERSE TRANSCRIPTION

Reverse transcription was performed with the RevertAid^TM^ H minus First strand cDNA synthesis kit (Fermentas Gmbh, Baden-Württemberg, Germany) in accordance to the manufacturer’s instructions. Briefly, a mixture of 1 μg RNA and 1 μl random hexamer primer solution was mixed first. Then, 8 μl of reaction mixture containing 4 μl of 5× reaction buffer (0.25 mol^-1^ Tris-HCl pH 8.3, 0.25 mol^-1^ KCl, 0.02 mol^-1^ MgCl_2_, 0.05 mol^-1^ DTT), 2 μl of 0.01 mol^-1^ dNTP mix, 20 units of ribonuclease inhibitor, 200 units of RevertAid^TM^ H minus M-MuLV Reverse Transcriptase was added. The reaction mixture was incubated for 5 min at 25°C followed by 60 min at 42°C. The reaction was terminated by heating at 70°C for 5 min and then cooled to 4°C. cDNA samples were checked by PCR and stored at -20°C for further use.

### REAL-TIME PCR

Real-time PCR was used to quantify the expression level of all the flagella-related genes and was performed with Maxima®; SYBR Green/ROX qPCR Master Mix (Fermentas, Fisher Scientific, Erembodegem, Belgium) as described previously (Yang and Defoirdt, 2014). The reaction was performed in an StepOne^TM^ Real-Time PCR System thermal cycler (Applied Biosystems, Gent, Belgium) in a total volume of 25 μl, containing 12.5 μl of 2× SYBR green master mix, 300 nM of forward and reverse primers and 2 μl of template cDNA. The thermal cycling consisted an initial denaturation at 95°C for 10 min followed by 40 cycles of denaturation at 95°C for 15 s and primer annealing and elongation at 60°C for 1 min. Dissociation curve analysis was performed to check for the amplification of untargeted fragments. Data acquisition was performed with the StepOne^TM^ Software.

### REAL-TIME PCR DATA ANALYSIS (2^–ΔΔC_t_^ METHOD)

The real-time PCR was validated by amplifying serial dilutions of cDNA synthesized from 1 μg of RNA isolated from bacterial samples. Serial dilutions of cDNA were amplified by real time PCR using gene specific primers. ΔC_t_ (average C_t_ value of target–average C_t_ value of *rpoA*) was calculated for the different dilutions and plotted against the cDNA concentration. The slope of the graph was almost equal to 0 for all of the target nine genes. Therefore, the amplification efficiency of reference and the target genes was considered to be equal. Based on this precondition, real-time PCR data were analyzed using the 2^-ΔΔC_t_^ method (Schmittgen and Livak, 2008). The expression of the target genes was normalized to the endogenous control (*rpoA*) by calculating ΔC_t_:

$$\Delta \,C_{t}=C_{t\,\mathrm{target}}-C_{t\,\;r\,p\,o\,A}$$

and expressed relative to a calibrator strain by calculating ΔΔC_t_:

Re⁡l⁢a⁢t⁢i⁢v⁢e⁢  exp⁡r⁢e⁢s⁢s⁢i⁢o⁢n=2−Δ⁢Δ⁢Ct

Strain BB120 without any treatments was used as acalibrator. The relative expression was then calculated as

$$\Delta \,\Delta \,C_{t}=\Delta \,C_{t\,}-C_{t\,\;\;c\,a\,l\,i\,b\,r\,a\,t\,i\,o\,r}$$

### AXENIC HATCHING OF BRINE SHRIMP LARVAE

Two hundred milligrams of high-quality hatching cysts of *Artemia franciscana* (EG®; Type; INVE Aquaculture, Baasrode, Belgium) were hydrated in 18 ml of filtersterilized tap water for 1 h. Sterile cysts were obtained by decapsulation based on the method described by Marques et al. (2004). Briefly, 660 μl of NaOH (32%) and 10 ml of NaOCl (50%) were added to the hydrated cyst suspension to facilitate decapsulation. The process was stopped after 2 min by adding 14 ml of Na_2_S_2_O_3_ (10 g L^-1^). Filtered (0.22 μm) aeration was provided during the reaction. The decapsulated cysts were washed with filtered (passed through 0.22-μm membrane filter) and autoclaved (moist heat at 121°C for 15 min) artificle seawater (containing 35 g l^-1^ of instant ocean synthetic sea salt, Aquarium Systems, Sarrebourg, France). The cysts were resuspended in a 50-ml tube containing 30 ml of filtered, autoclaved seawater and hatched for 28 h on a rotor (4 min^-1^) at 28°C with constant illumination (*c.* 2000 lux). The axenity of cysts was verified by inoculating one ml of culture water into 9 ml of Marine broth and incubating at 28°C for 24 h. After 28 h of hatching, batches of 30 larvae were counted and transferred to fresh, sterile 50-ml tubes containing 30 ml of filtered and autoclaved seawater. Finally, the tubes were returned to the rotor and kept at 28°C. All manipulations were performed in a laminar flow to maintain sterility of the cysts and larvae.

### BRINE SHRIMP CHALLENGE TEST

The effects of the catecholamines and antagonists on the virulence of *V. harveyi* were determined in a standardized challenge test with gnotobiotic brine shrimp larvae. *V. harveyi* was incubated with or without NE or Dopa (50 μM) and with or without antagonists, and cultures were washed with phosphate-buffered saline (pH 7.4) prior to inoculation into the brine shrimp rearing water at 10^5^ CFU ml^-1^. The challenge tests were performed as described by Defoirdt et al. (2005) with some modifications. A suspension of autoclaved LVS3 bacteria (Verschuere et al., 1999) in filtered and autoclaved seawater was added as feed at the start of the challenge test at 10^7^ cells ml^-1^ culture water. Brine shrimp cultures to which only autoclaved LVS3 bacteria were added as feed, were used as controls. The survival of the larvae was counted 48 h after the addition of the pathogens. Each treatment was carried out in quadruplicate and each experiment was repeated twice to verify the reproducibility. In each test, the sterility of the control treatments were checked at the end of the challenge by inoculating 1 ml of rearing water to 9 ml of Marine Broth and incubating the mixture for 2 days at 28°C.

### STATISTICAL ANALYSES

Data analysis was carried out using the SPSS statistical software (version 15). Log transformed gene expression data were analysed using independent samples *t-*tests. Unless stated otherwise, all other data were compared with one-way ANOVA, followed by Tukey’s *post hoc* test.

## RESULTS

### EFFECTS OF CATECHOLAMINES AND ANTAGONISTS ON THE SWIMMING MOTILITY OF *V. harveyi*

**FIGURE 1:**
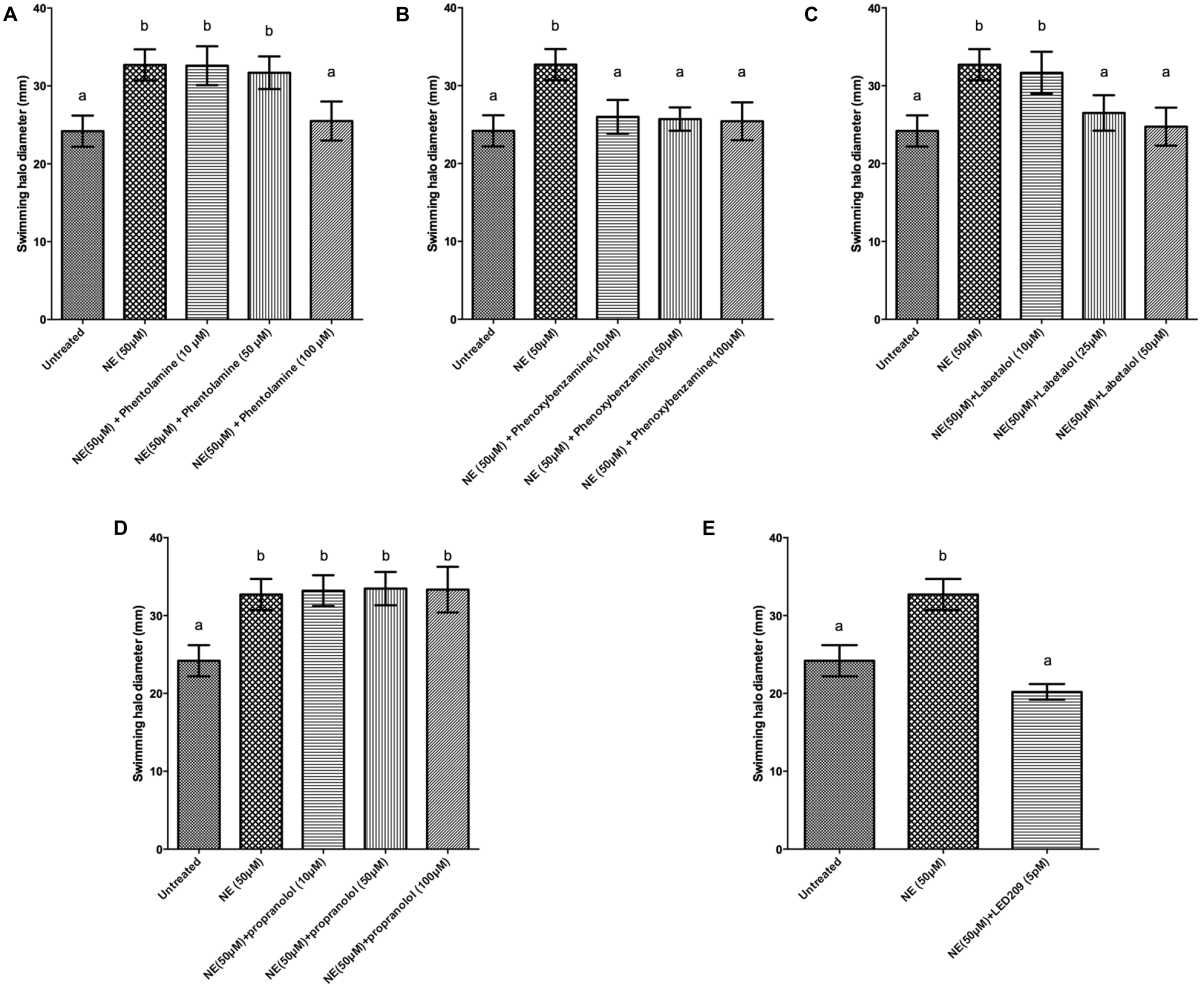
**Impact of norepinephrine (NE) and NE receptor antagonists on swimming motility of *V. harveyi.*** The error bars indicate the SD of six replicate cultures. Different letters indicate significant differences (One way ANOVA with Tukey’s *post hoc* test; *p* < 0.01). **(A)** NE with and without phentolamine, **(B)** NE with and without phenoxybenzamine, **(C)** NE with and without labetalol, **(D)** NE with and without propanolol and **(E)** NE with and without LED209.

**FIGURE 2:**
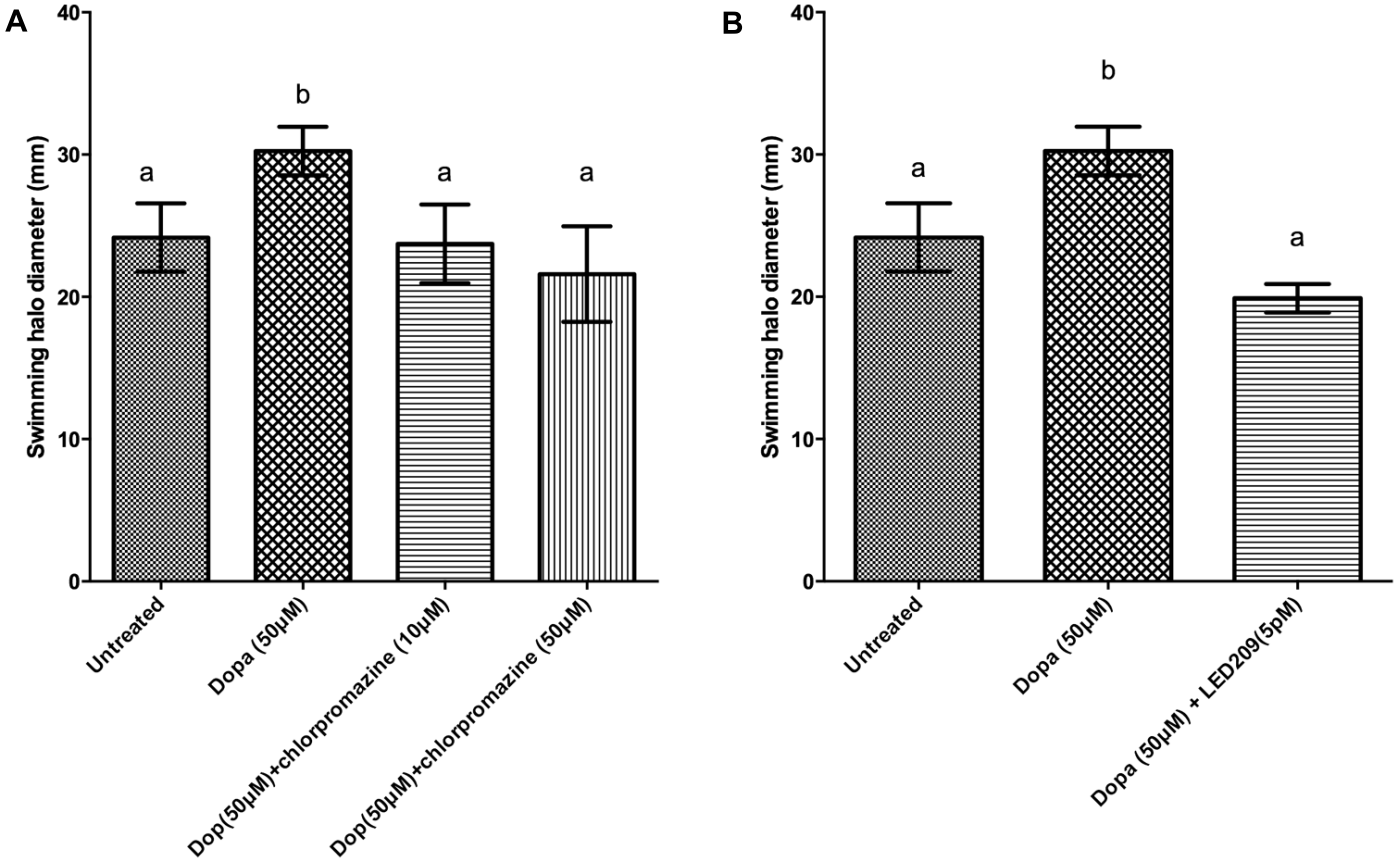
**Impact of dopamine (Dopa) and Dopa receptor antagonists on swimming motility of *V. harveyi.*** The error bars indicate the SD of six replicate cultures. Different letters indicate significant differences (One way ANOVA with Tukey’s *post hoc* test; *p* < 0.01). **(A)** Dopa with and without chlorpromazine and **(B)** Dopa with and without LED209.

Motility is required for the virulence of *V. harveyi* (Yang and Defoirdt, 2014), and since catecholamines are known to affect the motility of other pathogens (Lyte et al., 2011) we investigated the effect of catecholamines on the swimming motility of *V. harveyi* on soft agar. Both NE and Dopa could significantly increase the swimming motility of *V. harveyi* (**Figures 1** and **2**). We investigated the effects of catecholamines on the expression of ten selected genes involved in flagellar motility in *V. harveyi*, including six genes involved in the synthesis of the polar flagellum (both regulators and structural genes), two genes involved in the synthesis of lateral flagella, and two genes involved in chemotaxis. Both NE and Dopa significantly up-regulated the expressions of all selected genes (**Table 2**) which confirmed the stimulatory effect of catecholamines on flagellar motility.

**Table 2:** Impact of catecholamines on the expression of flagellar motility-related genes in *V. harveyi*.

Gene	Function	Relative expression (fold)^1^		
		Untreated	Norepinephrine (NE) (50 μM)	Dopamine (Dopa) (50 μM)
*flaA*	Polar flagellin	1.0 ± 0.1	2.8 ± 0.4**	1.6 ± 0.1**
*flaC*	Polar flagellin	1.0 ± 0.1	1.9 ± 0.1***	1.3 ± 0.2*
*flaK*	Polar flagellar regulator	1.0 ± 0.3	2.9 ± 0.2***	1.7 ± 0.3**
*fliA*	Polar flagellar biosynthesis sigma factor	1.0 ± 0.4	2.9 ± 0.5**	2.2 ± 0.1***
*fliS*	Polar flagellin specific chaperone	1.0 ± 0.3	3.2 ± 0.5**	1.7 ± 0.1***
*flgB*	Flagellar basal body rod	1.0 ± 0.2	3.2 ± 0.3**	1.4 ± 0.4**
*cheA*	Chemotaxis protein	1.0 ± 0.1	2.5 ± 0.3***	1.9 ± 0.2**
*cheR*	Chemotaxis protein	1.0 ± 0.4	2.0 ± 0.1**	1.4 ± 0.3*
*lafA*	Lateral flagellar flagellin	1.0 ± 0.3	3.0 ± 0.4**	1.9 ± 0.1***
*lafK*	Lateral flagellar regulator	1.0 ± 0.2	3.0 ± 0.6**	1.6 ± 0.4**

^1^For each gene, the expression in untreated cultures was set at 1 and the expression in cultures supplemented with catecholamines was normalized accordingly using the 2^-ΔΔC_*t*_^ method. Data are average ± SD of three *V. harveyi* cultures. Asterisks indicate a significant difference when compared to untreated *V. harveyi* (independent samples *t*-test; *p < 0.05; **p < 0.01; ***p < 0.001).

We further evaluated whether catecholamine receptor antagonists could neutralize catecholamine-induced swimming motility. The α-adrenergic antagonists phentolamine and phenoxybenzamine, the α-and β-adrenergic antagonist labetalol and the bacterial catecholamine receptor antagonist LED209 could neutralize NE-induced swimming motility (**Figure 1**), whereas the non-selective β-adrenergic receptor antagonist propranolol and the dopaminergic antagonist chlorpromazine had no effect (data not shown). Further, the dopaminergic antagonist chlorpromazine and the bacterial catecholamine receptor antagonist LED209 neutralized Dopa-induced motility (**Figure 2**), whereas adrenergic receptor antagonists had no effect (data not shown). Finally, we also checked the swimming motility of bacteria in the presence of only antagonists and equivalent volumes of the solvents as used in combination with the catecholamines, and no effects were observed (data not shown).

### EFFECTS OF CATECHOLAMINES AND ANTAGONISTS ON THE GROWTH OF *V. harveyi* IN SERUM-SUPPLEMENTED MEDIUM

**FIGURE 3:**
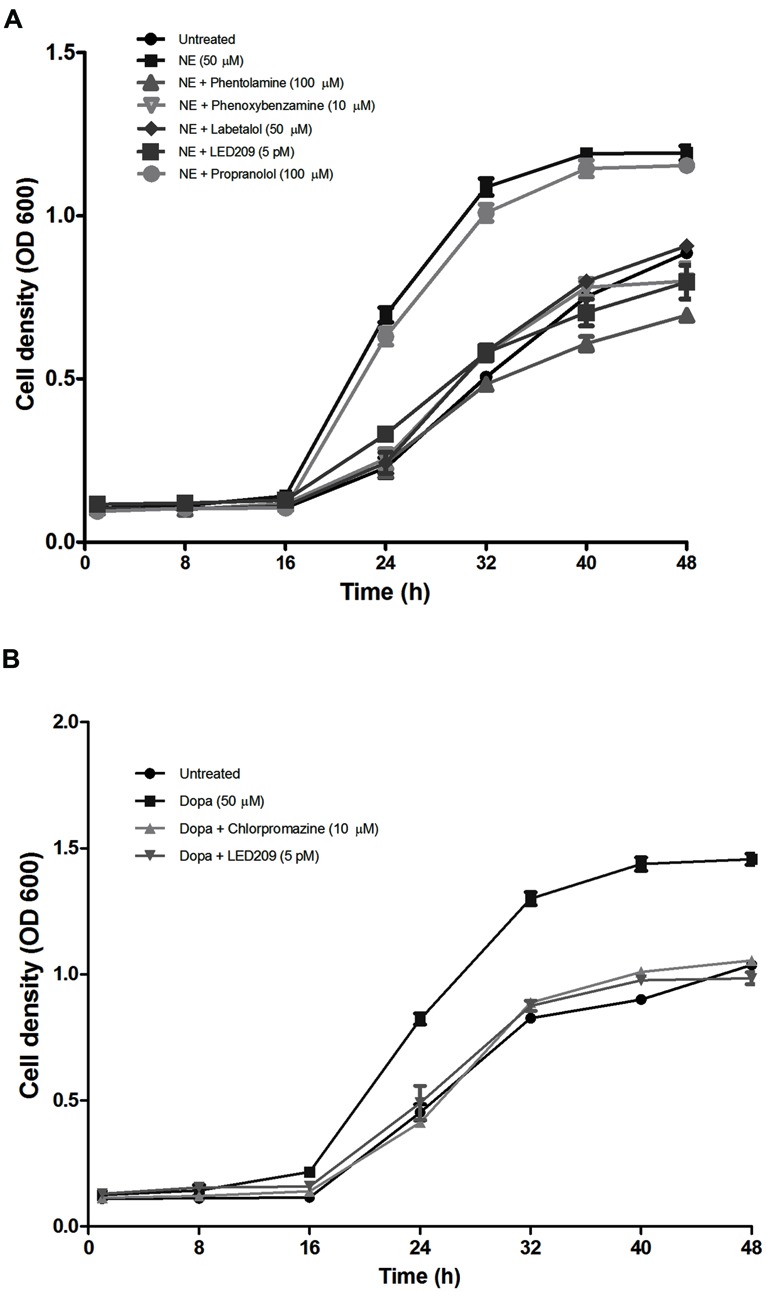
**Impact of catecholamines and catecholamine receptor antagonists on the growth of *V. harveyi* in LB_**35**_ broth containing 30% (v/v) serum. (A)** NE and adrenergic antagonists. **(B)** Dopa and dopaminergic antagonists. The initial density of *V. harveyi* was 10^2^ CFU/ml. Error bars represent the SD of three independent cultures.

The addition of NE or Dopa increased the growth of *V. harveyi* in Marine Broth containing 30% (v/v) serum (**Figure 3**) and resulted in a 2.4- and 2.1-fold increase in the growth rate of *V. harveyi*, respectively (0.59 ± 0.05 h^-1^ and 0.53 ± 0.04 h^-1^ in the presence of NE and Dopa, respectively, compared to 0.25 ± 0.03 h^-1^ for the untreated control). When compared to untreated cultures, the maximum turbidity was 1.6-fold higher for both NE and Dopa supplemented cultures. The catecholamines had no effect on growth of *V. harveyi* in medium without serum (data not shown).

In further experiments, we investigated whether catecholamine receptor antagonists could neutralize the growth-stimulatory effects of the catecholamines in serum-supplemented LB_35_ broth containing 50 μM catecholamines. The results were consistent with what we observed in the motility assays: antagonists with α-adrenergic activity (but not antagonists with only β-adrenergic activity) were able to inhibit NE-induced growth (**Figure 3A**), and the adrenergic antagonists were not able to neutralize Dopa-induced growth (data not shown). Further, the dopaminergic antagonist chlorpromazine neutralized Dopa-induced growth but had no effect on NE-induced growth (**Figure 3B**). The prokaryotic catecholamine receptor antagonist LED209 was able to neutralize the effects of both NE and Dopa. None of the antagonists and solvents used to dissolve the antagonists affected the growth of *V. harveyi* when tested alone at the same volumes as used in combination with catecholamines (data not shown), which indicates that the growth inhibition by the antagonists was not due to toxicity, but a specific antagonism of the bacterial response to catecholamines.

### EFFECTS OF CATECHOLAMINES AND ANTAGONISTS ON SIDEROPHORE PRODUCTION

**FIGURE 4:**
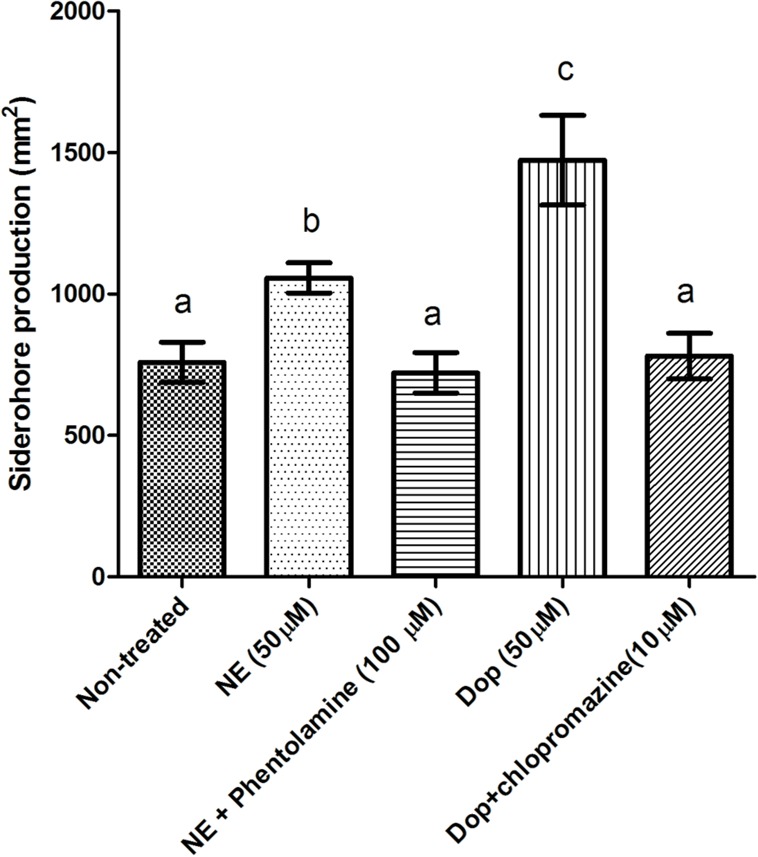
**Impact of catecholamines and catecholamine receptor antagonists on siderophore production by *V. harveyi.*** The concentration of catecholamines used is 50 μM. Phentolamine and chlorpromazine was added at a concentration of 100 and 10 μM, respectively. The error bars indicate the SD of six replicate cultures. Different letters indicate significant differences (One way ANOVA with Tukey’s *post hoc* test; *p* < 0.01).

In order to further substantiate the link between iron availability and growth induction by catecholamines in serum-supplemented medium, we determined the impact of the catecholamines on siderophore production. Both NE and Dopa significantly increased the siderophore production of *V. harveyi*, and the effect could be neutralized by phentolamine and chlorpromazine at a concentration of 100 and 10 μM, respectively (**Figure 4**). This is consistent with the antagonist concentrations needed to neutralize the growth-inducing effect of the catecholamines in serum-supplemented medium.

### EFFECTS OF CATECHOLAMINES AND ANTAGONISTS ON BIOFILM FORMATION AND EXOPOLYSACCHARIDE PRODUCTION

**FIGURE 5:**
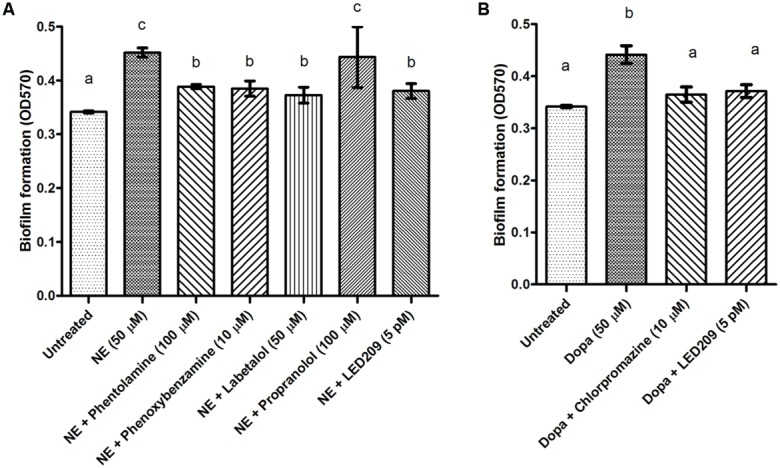
**Impact of catecholamines and catecholamine receptor antagonists on biofilm formation by *V. harveyi*. (A)** NE and adrenergic antagonists. **(B)** Dopa and dopaminergic antagonists. The error bars represent SD of three independent experiments. Different letters indicate significant differences (One way ANOVA with Tukey’s *post hoc* test; *p* < 0.01).

**FIGURE 6:**
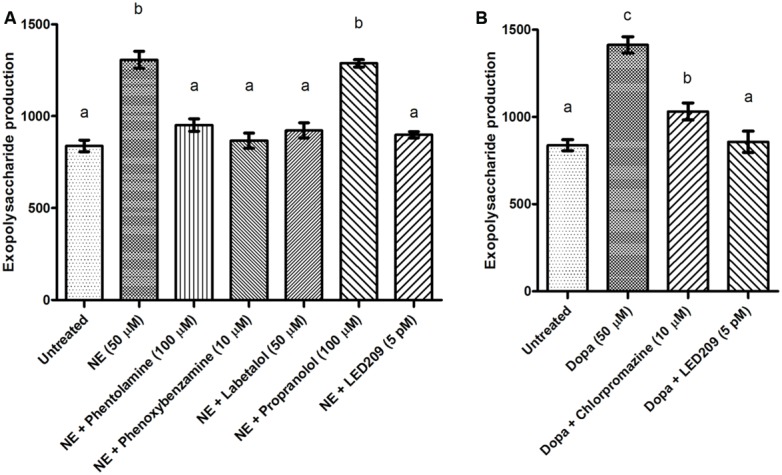
**Impact of catecholamines and catecholamine receptor antagonists on exopolysaccharide production of *V. harveyi*. (A)** NE and adrenergic antagonists. **(B)** Dopa and dopaminergic antagonists. The error bars represent SD of three independent experiments. Different letters indicate significant differences (One way ANOVA with Tukey’s *post hoc* test; *p* < 0.01).

Biofilm formation of *V. harveyi* was determined by crystal violet staining. The catecholamines significantly increased biofilm formation, and the effect was blocked by the antagonists (**Figure 5**). Because exopolysaccharide production is one of the major factors affecting biofilm formation, we also determined the impact of the catecholamines on exopolysaccharide production by Calcofluor white staining. The catecholamines significantly increased exopolysaccharide production (2.5- and 2.0-fold increase in the presence of NE and Dopa, respectively), and this could be neutralized by the antagonists (**Figure 6**). Importantly, the antagonists and solvents showed no influence on biofilm formation or exopolysaccharide production in the absence of catecholamines (data not shown).

### EFFECTS OF CATECHOLAMINES AND ANTAGONIST ON OTHER VIRULENCE FACTORS

The impacts of catecholamines on five other virulence factors (lipase, phospholipase, gelatinase, hemolysin, and caseinase production) were determined as well. NE and Dopa slightly decreased gelatinase activity (1.2- and 1.1-fold, respectively), while there was no significant effect on caseinase, lipase, phospholipase, or hemolytic activity (data not shown).

### EFFECTS OF CATECHOLAMINES AND ANTAGONIST ON THE VIRULENCE OF *V. harveyi* TOWARD BRINE SHRIMP LARVAE

*In vitro* experiments revealed that the catecholamines NE and Dopa positively regulated virulence-related phenotypes in *V. harveyi,* including growth in an iron-limited environment, swimming motility, biofilm formation, and exopolysaccharide production. To examine whether this results in an increased virulence of the bacterium *in vivo*, we performed a standardized challenge test with gnotobiotic brine shrimp larvae. In order to exclude any direct effects of the catecholamines on the host, *V. harveyi* was pretreated with the hormones for 4 h, after which the pathogen was washed with phosphate-buffered saline and then inoculated into the brine shrimp rearing water. Significantly higher brine shrimp mortality rates were observed when exposing *V. harveyi* to NE or Dopa prior to the challenge (**Table 3**).

**Table 3:** Impact of pretreatment of *V. harveyi* with catecholamines and catecholamine receptor antagonists on virulence of the bacterium toward gnotobiotic brine shrimp larvae.

Treatment	Survival (%)^a^
Control	100 ± 0^c^
*V. harveyi*	50 ± 5^b^
*V. harveyi* [50 μM NE]	27 ± 6^a^
*V. harveyi* [50 μM NE + 100 μM phentolamine]	48 ± 3^b^
*V. harveyi* [50 μM NE + 10 μM phenoxybenzamine]	47 ± 3^b^
*V. harveyi* [50 μM NE + 50 μM labetalol]	48 ± 3^b^
*V. harveyi* [50 μM NE + 100 μM propanolol]	29 ± 4^a^
*V. harveyi* [50 μM NE + 5 pM LED209]	55 ± 5^b^
*V. harveyi* [50 μM Dopa]	35 ± 5^a^
*V. harveyi* [50 μM Dopa + 50 μM chlorpromazine]	47 ± 3^b^
*V. harveyi* [50 μM Dopa + 5 pM LED209]	52 ± 8^b^

*^a^Survival after 2 days of challenge with *V. harveyi* ATCC BAA-1116 (average ± SE of four replicates). Survival in the control treatment was set at 100% and the other treatments were normalized accordingly. Square brackets refer to pretreatment – *V. harveyi* was either or not pretreated with catecholamines and antagonists for 4 h and washed prior to inoculation into the brine shrimp rearing water. Values with a different superscript letter are significantly different from each other (One way ANOVA with Tukey’s *post hoc* test; *p* < 0.01).*

We further evaluated the effects of catecholamine antagonists by pretreating *V. harveyi* with both catecholamines and antagonists. The α-adrenergic antagonists phentolamine and phenoxybenzamine, the α- and β-adrenergic antagonist labetalol and the prokaryotic catecholamine receptor antagonist LED209 were all able to neutralize the increased virulence induced by NE, whereas the dopaminergic antagonist chlorpromazine and LED209 neutralized the effect of Dopa (**Table 3**). Pretreatment of *V. harveyi* with the antagonists had no effect on virulence in the absence of catecholamines (data not shown).

## DISCUSSION

The successful interaction between bacteria and their host depends not only on a coordinated response to population density, temperature and pH (Mekalanos, 1992; Winzer and Williams, 2001), but also on the detection of diverse host cell effector molecules such as catecholamine stress hormones (Burton et al., 2002). Exposure of bacteria to catecholamine stress hormones has been demonstrated to stimulate both growth and production of virulence-related factors in various pathogens of terrestrial animals and humans, such as *E. coli* (Bansal et al., 2007), *C. jejuni* (Cogan et al., 2007), *S. typhimurium* (Bearson and Bearson, 2008), and *V. parahaemolyticus* (Nakano et al., 2007b). The present study demonstrates that the catecholamines NE and Dopa could significantly increase the production of major virulence factors and growth in serum-supplemented medium of *V. harveyi*, a major pathogen of aquatic organisms with a broad host spectrum (both vertebrates such as fish and invertebrates such as crustaceans and mollusks). This result is in good agreement with, and considerably extends, the previous reports.

The catecholamines significantly increased the virulence of *V. harveyi* toward gnotobiotic brine shrimp larvae. The pathogen was pretreated with catecholamines in order to avoid a direct effect of catecholamines on the host (e.g., decreased activity of the defense system) and to ensure that any impact on survival of challenged larvae was due to increased virulence of the pathogen. This is further substantiated by our observation that in addition to their effect on growth in serum-supplemented medium and siderophore production, the catecholamines increased the production of several other phenotypes that are important for infection. Indeed, both NE and Dopa significantly stimulated biofilm formation, exopolysaccharide production, swimming motility, and the expression of genes involved in flagellum synthesis (structural genes and regulators including the flagellar master regulator) in *V. harveyi*. These results agree well with previous reports documenting the impact of catecholamines on biofilm formation in *Staphylococcus epidermidis* (Lyte et al., 2003) and on swimming motility in *E. coli*, *C. jejuni,* and *Edwardsiella tarda* (Cogan et al., 2007; Kendall et al., 2007; Wang et al., 2011).

Catecholamines are produced by hemocytes of invertebrates, and concentrations in the hemolymph of shrimp and prawn that have been reported range between 10 and ∼3 μM (Chen et al., 2003; Hsieh et al., 2006; Pan et al., 2014). These concentrations are lower than the concentrations used in this study. However, local concentrations can be considerably higher. For instance, the intrasynaptic concentration of NE in the central nervous system of mammals is as high as 10 mM (versus nM levels in serum; Lyte, 2004). Hence, upon infection, pathogens can come into contact with local concentrations that are several orders of magnitude higher than those that are found in hemolymph (or serum in case of vertebrates). This probably is also the case when tissues and/or hemocytes are damaged during infection and hence, elevated catecholamine levels might be a cue informing the pathogen of tissue damage.

Pretreatment of *V. harveyi* with catecholamines simulated transmission of the pathogen from a host site showing elevated catecholamine levels (e.g., due to cell or tissue damage). In combination with our observation that the catecholamines increased biofilm formation, exopolysaccharide production, and swimming motility of *V. harveyi* (which are all important during the initial stages of infection), our results suggest that catecholamine sensing increases the success of transmission to a new host. Hence, elevated catecholamine levels might be a cue informing the pathogen that the infection reached a final stage (cell and tissue damage) and that it is time to leave the host.

Catecholamine receptor antagonists have been used extensively to identify and characterize catecholamine receptors in mammals. The effects of various antagonists on growth and production of virulence factors in *V. harveyi* have been investigated in the present study, and the results demonstrated that α- but not β-adrenergic receptor antagonists could block responses to NE, but did not show any effect on Dopa responsiveness. On the contrary, dopaminergic receptor antagonists neutralized induction caused by Dopa, but did not neutralize induction by NE. Similar results have been reported in enteric pathogens of terrestrial animals (Freestone et al., 2007), and suggest that bacterial response systems for catecholamines possess a degree of specificity similar to mammalian catecholamine receptors. In contrast to the wealth of adrenergic and dopaminergic receptors described in eukaryotes, there have been only few reports examining the presence of such receptors in bacteria. Clarke et al. (2006) reported that NE was able to be recognized by the *E. coli* O157:H7 two-component regulator sensor kinase QseC in *in vitro* constructs, leading to the hypothesis that this is the bacterial catecholamine receptor. Later on, a QseC antagonist, LED209, has been identified in a high-throughput screen (Rasko et al., 2008). We found that LED209 was able to neutralize the effects of both NE and Dopa, indicating that a similar response system for catecholamines might exist in *V. harveyi*. This is also substantiated by the fact that the *V. harveyi* ATCC BAA-1116 genome contains a QseC homolog. Alternatively, since our experiments with eukaryotic catecholamine receptor antagonists showed that dopaminergic antagonists did not neutralize NE-induced effects and vice versa, there are probably at least two different catecholamine receptors in *V. harveyi*, with LED209 being able to bind to both of them. Further research will be needed in order to identify the catecholamine receptors and the signal transduction pathways in *V. harveyi.*

Both NE and Dopa significantly stimulated the growth of *V. harveyi* in serum-supplemented medium, and this effect could be neutralized by eukaryotic catecholamine receptor antagonists. Similar results have been reported in other Gram-negative bacteria such as *E. coli* and *V. parahaemolyticus*, and the stimulation of growth in serum-supplemented medium by catecholamines has mainly been attributed to their ability to facilitate iron removal from the host iron-binding proteins transferrin and lactoferrin (Lyte, 2004; Nakano et al., 2007b). We used bovine serum since it was practically not feasible to obtain sufficient amounts of crustacean serum. However, it should be noted that crustaceans produce equivalents of iron-binding proteins produced by mammals (e.g., Toe et al., 2012). Our observations that catecholamine receptor antagonists are able to neutralize the growth-stimulatory effect of catecholamines and that adrenergic receptor antagonists were found to show no effect on Dopa-induced growth and vice versa suggest that a regulatory mechanism involving catecholamine receptors is involved as well, and this has also been reported for enteric pathogens such as *E. coli* O157:H7, *S. enterica* and *Y. enterocolitica* (Freestone et al., 2007). To confirm the hypothesis that in addition to increasing iron uptake in a direct way, catecholamines induced an iron uptake mechanism, we investigated the effect of catecholamines on siderophore production, and found that both NE and Dopa could significantly induce siderophore production. Siderophores have been reported to be essential for catecholamine-induced growth in other Gram-negative bacteria such as *E. coli* and *Salmonella*, where they serve to internalize the iron removed by the catecholamines (Freestone et al., 2003; Williams et al., 2006). Finally, it should be noted that in addition to limiting iron availability, serum might have other effects on the pathogens as well. Serum, e.g., contains proteins (some of which might have antimicrobial activity; Zasloff, 2002). However, in view of the literature that is available on other pathogens and our observation that catecholamines increase siderophore production we think that modulation of iron availability is the most plausible explanation of growth stimulation by catecholamines in the presence of serum.

## CONCLUSION

Our study has shown that the catecholamines NE and Dopa increase the virulence of *V. harveyi* by increasing swimming motility, biofilm formation, exopolysaccharide production, and growth in environments with low iron availability, and the effects could be neutralized by antagonists for eukaryotic catecholamine receptors and the bacterial catecholamine receptor antagonist LED209. Different effects of the adrenergic and dopaminergic receptor antagonists indicate the presence of specific sensing systems for different catecholamines in *V. harveyi*.

## Conflict of Interest Statement

The authors declare that the research was conducted in the absence of any commercial or financial relationships that could be construed as a potential conflict of interest.
